# Using self‐determination theory in research and evaluation in primary care

**DOI:** 10.1111/hex.13620

**Published:** 2022-10-01

**Authors:** Huayi Huang, Harry H. X. Wang, Eddie Donaghy, David Henderson, Stewart W. Mercer

**Affiliations:** ^1^ Usher Institute Centre for Population Health Sciences University of Edinburgh Edinburgh UK; ^2^ School of Public Health Sun Yat‐Sen University Guangzhou Guangdong Sheng China

**Keywords:** motivation, personal autonomy, primary care, professional autonomy, self‐determination theory

## Abstract

**Background:**

Multimorbidity (the co‐existence of two or more long‐term conditions within an individual) is a complex management challenge, with a very limited evidence base. Theories can help in the design and operationalization of complex interventions.

**Objective:**

This article proposes self‐determination theory (SDT) as a candidate theory for the development and evaluation of interventions in multimorbidity.

**Methods:**

We provide an overview of SDT, its use in research to date, and its potential utility in complex interventions for patients with multimorbidity based on the new MRC framework.

**Results:**

SDT‐based interventions have mainly focused on health behaviour change in the primary prevention of disease, with limited use in primary care and chronic conditions management. However, SDT may be a useful candidate theory in informing complex intervention development and evaluation, both in randomized controlled trials and in evaluations of ‘natural experiments’. We illustrate how it could be used multimorbidity interventions in primary care by drawing on the example of CARE Plus (a primary care‐based complex intervention for patients with multimorbidity in deprived areas of Scotland).

**Conclusions:**

SDT may have utility in both the design and evaluation of complex interventions for multimorbidity. Further research is required to establish its usefulness, and limitations, compared with other candidate theories.

**Patient or Public Contribution:**

Our funded research programme, of which this paper is an early output, has a newly embedded patient and public involvement group of four members with lived experience of long‐term conditions and/or of being informal carers. They read and commented on the draft manuscript and made useful suggestions on the text. They will be fully involved at all stages in the rest of the programme of research.

## 1 INTRODUCTION

Multimorbidity is usually defined as the co‐existence of two or more long‐term conditions within an individual.^1^ Multimorbidity presents complex challenges at the level of the individual (patient), family, healthcare team, healthcare system, policymakers and healthcare planners.^2^ For patients, multimorbidity increase mortality, reduces the quality of life and impacts roles and responsibilities.^1^, ^2^, ^3^ Multimorbidity can place a burden on families, and in countries without universal coverage, can be financially catastrophic in terms of loss of earnings and healthcare costs.^4^, ^5^ Multimorbidity increases the use of health services, presenting a challenge to policymakers and healthcare planners, who have traditionally invested more in secondary care than primary care, and taken a single‐disease or single‐bodily system approach to care, leading to fragmentation of care for patients and burgeoning costs to the system due to the multiple clinical specialities that a patient with multimorbidity may be referred to.^1^, ^2^, ^3^, ^4^, ^6^, ^7^

The evidence base for how best to treat patients with multimorbidity is very limited, and most interventions to date have not been evaluated or demonstrated effectiveness or cost‐effectiveness.^8^ It is widely accepted that interventions in multimorbidity are likely to be complex,^9^ and expert guidelines exist on the development and evaluation of complex interventions.^9^, ^10^ A recent Delphi study funded by the MRC‐NIHR Methodology Research Panel also reached a consensus that theory is a crucial part of developing complex interventions and concluded that a theory‐driven approach to intervention development and evaluation is more likely to be effective than a purely pragmatic or empirical approach.^11^ Thus, theories can help illuminate and clarify the processes of change expected, and how these are likely to be achieved through the intervention. It is therefore of interest that a recent review of multimorbidity interventions found that theory was often absent from interventions in healthcare settings.^12^ The newly updated MRC guidance on complex interventions considers two approaches—developing a new intervention or evaluating an intervention that already exists, and the importance of theory is highlighted, as it was in the original guidelines^13^ and subsequent revisions.^14^

In the current article, we focus on one particular theory, self‐determination theory (SDT), which we propose as a candidate theory in the development and evaluation of interventions in multimorbidity in primary care. Daily self‐management (lifestyle) in relation to chronic conditions in general and multimorbidity, in particular, is obviously important and SDT seems intuitively suited to self‐management support. We briefly explain SDT, and review its recent use in health contexts in general and specifically in complex interventions in primary care, before going on to illustrate how it could be used in interventions in multimorbidity in primary care.

## 2 METHODS

### 2.1 Review of SDT literature

For our overview of SDT, we have drawn on the original work by Deci and Ryan,^15^ the proposers of the theory, and the information on the Centre for Self‐Determination Theory website (https://selfdeterminationtheory.org/).

For an overview of the recent use of SDT in studies in health contexts, we searched for published reviews in the last 6 years that had focused on SDT exclusively. Our sources, search terms, inclusion and exclusion criteria and PRISMA flow chart are shown in Supporting Information: Table S1.

For our rapid review of SDT in complex interventions in primary care, we searched 12 bibliographical databases as available through the University of Edinburgh; no date limits were set for this as we expected few papers and wanted to try to ensure we did not miss any relevant studies (see Supporting Information: Table S2 for further details).

For both rapid reviews, H. H. conducted the searches and screened the titles and abstracts, and H. H. and S. W. M. read the full papers of the identified papers after screening and reached an agreement on which papers to include through discussion.

In considering primary care specifically, we use the definition of primary care proposed by the National Institute for Clinical Effectiveness (NICE): ‘Primary care is healthcare delivered outside hospitals. It includes a range of services provided by GPs, nurses, health visitors, midwives and other healthcare professionals and allied health professionals such as dentists, pharmacists and opticians. It includes community clinics, health centres and walk‐in centres’ (https://www.nice.org.uk/Glossary?letter=P).

## 3 FINDINGS

### 3.1 An overview of SDT

SDT relates to the extent to which human behaviour is self‐motivated and self‐determined, and is based on the proposal that there are three basic psychological needs that must be satisfied if individuals are to achieve health and well‐being.^15^, ^16^, ^17^ These are; autonomy (volition—a sense that one has choices), relatedness (a sense of belongingness and connectedness with others) and competence (a sense of mastery and effectiveness). According to SDT, the satisfaction of these three basic psychological needs fosters intrinsic motivation (the natural, inherent drive in human beings to thrive). These three basic psychological needs are regarded as *innate* tendencies in human development, as described by the originators of the theory, Ryan and Deci; ‘well‐being is like a three‐legged stool; pull out any one of these supports and the stool will fall’.^16^,p.250

**Figure 1:**
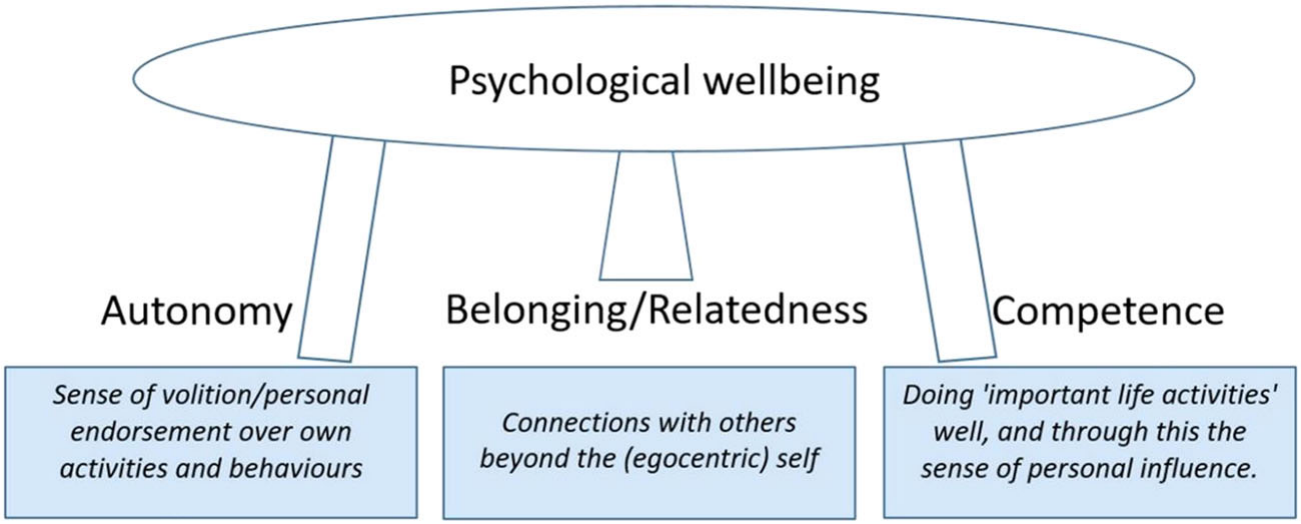
The three basic ingredients of mental well‐being according to SDT. SDT, self‐determination theory.

Motivation that is entirely extrinsic (driven by external demands or rewards) is the least self‐determined form. However, in reality, motivation for most people is on a spectrum from intrinsic to extrinsic. SDT postulates that interventions that encourage individuals towards a more intrinsic form of motivation will lead to better health behaviours and outcomes by satisfying the three basic psychological needs (Figure 1).

**Figure 2:**
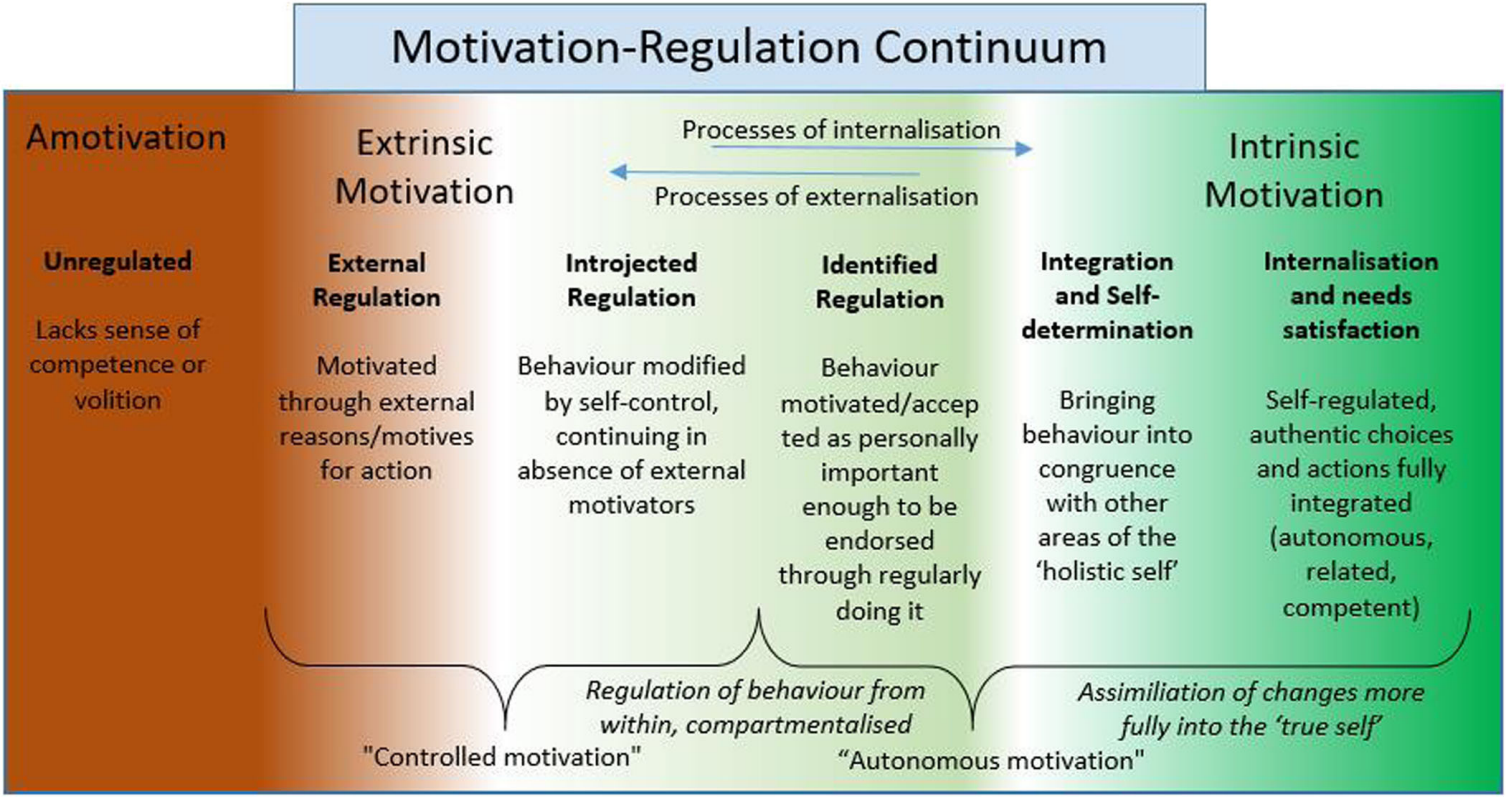
How external motives for action, become increasingly internalized into the ‘autonomous self’

SDT thus argues that satisfaction of these three basic ‘growth needs’ is a pre‐requisite for adaptive, ‘healthy’ changes to take place—leading to increasing *integration* and *internalization* of such changes along the motivation‐regulation continuum—as shown in Figure 2 and explained further in Table 1.

**Table 1:** A brief explanation of levels of quality in the motivation‐regulation continuum

Levels of motivation quality (ascending)	Brief summary
*External regulation* (Controlled motivation)	Regulation due to reasons for action seen as external to the self.
*Introjected regulation* (Controlled motivation)	Limited internalization of reasons for action, but regulatory patterns still significantly conditional on external feedback; these reasons for action remain poorly integrated with the true self, and are at risk of conflict/incongruence with it.
*Identified regulation* (Autonomous motivation)	Hypothetical quote, where a participant in this kind of regulation says: ‘*This* is just what I often do’
*Integrated regulation and self determination* (Autonomous motivation)	Hypothetical quote: ‘What I do is *this* [action], and *this is* part of the real me’
*Intrinsic regulation of the self, internalization and needs satisfaction* (Autonomous motivation)	Reasons for action are associated strongly with the sense of ‘true self’, with the individual for example sustaining a long‐term relationship with a ‘bigger cause’ of value for others as well as the self (e.g., enabling healthier lives). A state of true self‐regulation simultaneously satisfies the three basic psychological needs described in Figure 1 (Autonomy, Relatedness and Competence), and is generative of optimal psychological health.

*Note*: Amotivation is simply a state in which no action takes place (e.g., due to a poor sense of volition or competence).

It is important to stress that SDT postulates that, given the right opportunities and conditions, human beings naturally seek competence, relatedness and autonomy^15^, ^16^, ^18^ and that such internal motivation is *autonomous* (Figure 2, right), that is, when people willingly engage in healthy activities simply for personal enjoyment and interest—it is part of ‘who they are’. For example, certain children enjoy running simply for the pleasure of running—and as adults may continue this activity because its what they have always done, and they still derive immense pleasure from it. Those individuals at the other end of the motivation continuum lack such autonomous motivation (Figure 2, left) either because they perceive that they are lacking competence in the activity or they get no intrinsic pleasure from doing it (‘amotivation’), or because the motives for certain activities and behaviours originate from external sources (‘external regulation’). For example, the schoolboy who is forced to run by the Physical Education teacher, and simply does it because he will be punished if he does not, is unlikely to continue running activities of his own choice when he becomes older. However, such extrinsic motivation is not necessarily static, and people can transition from the left to right in Figure 2, with the right support and circumstances. When ‘motives for action’ move from more external to more internalized and thus more integral to their ‘true whole self’ (Figure 2, right), human beings experience the highest quality of self‐determination and psychological health and well‐being. Individuals who regularly experience satisfaction with their sense of autonomy, competence and relatedness then tend to become more self‐determined in their choices and actions. As the originally extrinsic motives and reasons for activities become more internalized and integrated into the ‘self’ of an individual, they become more intrinsic and assimilated into self‐identity and self‐regulation, and are experienced typically as autonomous rather than controlled forms of motivation (Figure 2, right).

To summarize, in the words of Deci and colleagues,^15^, ^16^, ^18^ the proposers of SDT;

‘There are three basic psychological needs, the satisfaction of which is essential to optimal development, integrity and well‐being. These are the needs for autonomy, competence and relatedness. Failure to satisfy any of these needs will be manifested in diminished growth, integrity and wellness. In addition, need frustration, typically due to the thwarting of these basic needs, is associated with greater ill‐being and more impoverished functioning’. And;

‘The competence, autonomy, and relatedness needs, for example, make clear what people need to do in order to be healthy—for example, do important activities well, endorse their actions, and connect with others’.

*Beneficence/benevolence* has recently been proposed as a fourth fundamental need for SDT,^19^ and has been operationalized so far as being about positively and pro‐socially contributing to the welfare of others, within one's social circle and in wider society. Emerging quantitative evidence suggests a robust association between this sense of beneficence, and enhancements in *well‐being* and *meaningfulness of work* ^19^, ^20^; but empirical research has yet to show that deprivation or frustration of opportunities to be benevolent, damages well‐being or predicts ill‐being for the benefactor. For a candidate construct to count as a ‘basic psychological need’, Ryan and Deci^16^,p.251 argue that there must be evidence that deprivation of opportunities for satisfying it is also damaging to well‐being. This is a criterion met by the constructs of autonomy, relatedness and competence; but not yet by evidence on the idea of benevolence as a similarly ‘basic’ psychological need. For this reason, we have not included it in the case study below.

### 3.2 Recent reviews of SDT in health contexts

We found 11 recent reviews of the use of SDT in health contexts,^21^, ^22^, ^23^, ^24^, ^25^, ^26^, ^27^, ^28^, ^29^, ^30^, ^31^, ^32^ which we summarize briefly below, starting with more general reviews of health behaviour change, and moving to reviews that have a more specific context, including those that have focused on particular medical conditions.

Ntoumanis et al.^21^ performed a meta‐analysis of over 70 studies of SDT‐informed intervention studies covering a wide range of health behaviours mainly concerning the primary prevention of disease. Although most reported positive changes, the effect sizes were noted to be modest and heterogeneous.

Gillison et al.^22^ conducted a systematic review and meta‐analysis of techniques to promote motivation for health behaviour change from an SDT perspective and identified 74 studies, most (80%) of which were randomised controlled trials (RCTs). Meta‐regression analysis showed that individual strategies had limited independent impact on outcomes, suggesting that such interventions require multiple co‐acting techniques.

Smith and Williams^23^ reviewed factors influencing motivation for change in clinical practice in different healthcare settings based around SDT and found that the closer an implementation process is to the autonomous motivation end of the continuum, the greater the willingness of staff to change their behaviour and the greater the likelihood of a successful and sustained outcome.

Tang et al.^24^ conducted a systematic review and meta‐analysis on 23 studies that examined SDT and well‐being in later life and found that basic psychological need satisfaction and more autonomous motivation were positively associated with well‐being. All studies considered satisfaction of the three basic psychological needs for competence, autonomy and relatedness as essential in predicting the quality of caregivers' motivation and thereby their well‐being. In this review, autonomous motivation was the most important determinant of caregivers' well‐being.

Dombestein et al.^25^ conducted an integrative review of SDT and informal care‐giver's motivation and found that satisfaction of the three basic psychological needs was essential in predicting the quality of caregivers' motivation with autonomous motivation being the most important determinant of caregivers' well‐being.

In specific medical conditions, Phillips and Guarnaccia^26^ conducted a systematic review of SDT‐based interventions for type 2 Diabetes prevention and treatment. The results were mixed and of variable quality, but the majority of the interventions resulted in health benefits. Kusec et al.^27^ conducted a narrative review to examine motivation in brain injury through an SDT lens and suggested that both intrinsic and extrinsic motivation may be important for change after brain injury.

Exercise has been a major focus of SDT‐based interventions. Saugy et al.^28^ reviewed research in physical education with a self‐determination framework, and Szabo and Juwono^29^ reviewed the efficacy of SDT‐based interventions in increasing students' physical activity, and both reviews suggest that SDT‐based interventions have the potential to increase physical activity. A review of pre‐school self‐regulation interventions from an SDT perspective found that targeting competence and nurturing children's autonomy led to more effective interventions, whereas relatedness appeared to have less impact.^30^

Finally, it is noteworthy that we identified only one systematic review of qualitative studies on the views of patients with chronic diseases which used an SDT perspective,^31^ which found only six studies. Most of these focused on the clinical aspects of managing a chronic condition and changing patient health behaviours, rather than the psychological and emotional needs of living with a chronic illness.

To summarize then, despite a large number of studies of SDT in health contexts there has been, as far as we can glean from these recent reviews, a limited focus on chronic conditions (mainly focused on diabetes) with no reviews identified that targeted patients with multimorbidity.

### 3.3 Studies using SDT in complex interventions in primary care

Our rapid review of SDT in complex interventions in primary care found only seven publications from four studies. In the first study, Hurley et al.^32^, ^33^, ^34^, ^35^ have published four papers from their study on ‘Self‐management of Osteoarthritis and Low back pain through Activity and Skills’ (SOLAS), which was a theory‐driven complex SDT intervention of self‐management of osteoarthritis and low back pain in primary care. This included a protocol for their cluster RCT feasibility trial,^32^ intervention development,^33^ views of the physiotherapists who delivered the intervention on the training programme^34^ and the findings of the cluster RCT feasibility trial,^35^ which found the intervention to be acceptable, with small improvements in some secondary outcomes at 2 and 6 months. However, recruitment of primary care centres and patients was problematic and the authors concluded that progression to a definitive trial would not be feasible.^35^ In this study, the authors used a theoretical domains framework to consider a range of behaviour change theories in the intervention mapping activities, before selecting SDT.^33^

The second study was a complex intervention in the general practice of social prescribing—the Glasgow ‘Deep‐End’ Community Link Worker Project (co‐led by S. W. M.)—conducted as a quasi‐experimental cluster RCT.^36^ It was not targeted at patients with multimorbidity, though most recruited did have multimorbidity spanning mental, physical and social problems.^37^ SDT was not used to design or quantitatively evaluate the intervention. Overall, patient outcomes did not improve, except for those who frequently engaged with the link workers and available community resources,^37^ and less than half of the practices fully engaged with the programme.^38^ A secondary analysis explored the utility of SDT in explaining the reported impact of social prescribing on 12 patients who had been qualitatively interviewed and found that patients who reported improvements in daily life also described the satisfaction of the three psychological needs and described changes toward more intrinsic regulation of behaviour following the intervention.^39^

The third study was by Bhatti et al.^40^ who used SDT to understand the social prescribing process in a qualitative study involving 18 focus groups involving 88 patients, plus 8 in‐depth one‐to‐one interviews. In this study, SDT was used as the theoretical framework for thematic analysis. They found that participants engaging in the social prescribing pathway in a community healthcare setting, broadly satisfied the elements present in SDT and that patients reported a range of positive outcomes from the intervention.

The fourth study identified was the CARE Plus study, which was led by S. W. M., and is explored in detail below as an example of how SDT may be used in the evaluation as well as in designing a definitive trial.

### 3.4 Using SDT in developing and evaluating complex interventions in multimorbidity—The example of the CARE Plus study

This study was a programme of research that developed a primary care‐based complex intervention (CARE Plus) for patients with multimorbidity living in areas of high socioeconomic deprivation in Scotland.^41^, ^42^ The intervention aimed to improve the quality of life and well‐being of patients aged 30–64 years, by experimentally ‘reversing’ the inverse care law.^43^ Consultations between general practitioners (GPs) and patients in primary care in deprived areas are hindered by a mismatch of need and supply; patients have high levels of complex multimorbidity, spanning mental, physical and social problems, and thus consult with complex problems.^44^ However, because there are too few GPs to meet these unmet healthcare needs, consultations are shorter, less patient‐centred, less enabling and have poorer outcomes than similar consultations in more affluent areas.^44^, ^45^, ^46^

The intervention developed in accordance with the MRC Complex Intervention Development Guidelines available at the time^14^, ^15^ which included identifying the target population through epidemiological work,^2^ the impact of multimorbidity in deprived areas,^47^, ^48^ understanding the challenges of managing multimorbidity that patients and practitioners face in deprived areas,^49^, ^50^ developing and optimizing the intervention in pilot studies,^41^ and testing its feasibility in Phase 2 exploratory cluster randomized controlled trial.^42^

The CARE Plus intervention consisted of longer consultations for targeted multimorbid patients, continuity of care, training and support for practitioners in delivering empathic, patient‐centred care and self‐management support materials for the patients.^42^ It had a cluster RCT design, with four practices receiving the complex intervention and four delivering usual care, with 76 patients in each arm of the trial and follow‐up at 6 and 12 months.^42^ The exploratory RCT was successful in showing the feasibility of the intervention, with evidence of likely effectiveness and cost‐effectiveness.^42^

The development of the CARE Plus intervention was informed by the available evidence base at the time but did not use a specific theory in its design.^41^, ^42^ However, after conducting the Phase 2 trial, a post hoc analysis was taken to analyse qualitative interviews with patients in this study, based on SDT, to explore if this could help explain why some patients had good outcomes and others did not.^51^ Out of the 14 patients interviewed, 6 reported changes in well‐being that improved daily life, 3 reported slight improvement (not impacting daily life) and 5 reported no improvement. Satisfaction of relatedness, competence and autonomy needs to be featured strongly in those reporting improved well‐being in daily life and this was also reflected in changes in self‐determined motivational regulation towards more intrinsic motivation. Satisfaction of basic needs and changes in motivation were not seen in those with little or no improvement in well‐being.^51^

Based on these findings, and the growing literature on the utility of SDT in people with long‐term conditions as discussed above, we outline below how the CARE Plus intervention could be further developed through SDT in preparation for a definitive Phase 3 cluster RCT by using SDT. In terms of the theoretical underpinning of an intervention, the new MRC guidelines recommend the use of programme theory^9^ and in the case of CARE Plus, SDT could be used as the core of the programme theory, by hypothesizing that improvements in outcomes will depend on basic needs satisfaction and a shift in patient motivation towards more intrinsic forms. We could then use SDT in further developing the intervention itself, by placing it at the centre of the patient‐centred approach and training primary care professionals to employ it, that is, by building a care plan and ongoing self‐management support by identifying with the patient goals based on changes that incorporate more intrinsic motivation, and which would support their basic psychological needs. Uncovering and supporting such goals will require an empathic, patient‐centred approach, sufficient time in the consultations and continuity of care.

**Figure 3:**
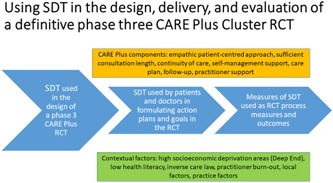
Using SDT in the design, delivery and evaluation of a definitive Phase 3 CARE Plus RCT. SDT, self‐determination theory.

For evaluation of the trial, SDT could be employed in both a process evaluation (in qualitative interviews) and as measured outcomes (measuring changes in basic need satisfaction and motivation). The CARE Plus ingredients, that are necessary to support the delivery of the intervention ‘wrap around’ the use of SDT to improve patient well‐being and quality of life. In addition, it is important to consider the contextual factors that currently thwart attempts to improve such patient outcomes and to be mindful of these in terms of the implementation of the intervention as well as in the evaluation of effectiveness (Figure 3).

## 4 DISCUSSION

In this article we have described the challenges of multimorbidity, the need for effective complex interventions in this area, and the importance of theory in complex intervention development and evaluation, with a focus on SDT. We have described SDT, and briefly reviewed how it has been used in studies to date and found that there has been very limited research on SDT in complex interventions, in primary care, in chronic diseases in general, and in multimorbidity specifically. We have used the example of the CARE Plus study to demonstrate how SDT can be used in primary care‐based research of complex interventions in patients with multimorbidity, illustrating its use in post hoc analysis, as well as (in the case of preparing CARE Plus for a definitive trial) as a central part of programme theory, training, process evaluation and outcomes.

We propose that SDT may be a useful theory in both ongoing interventions (already implemented by policymakers, such as the Deep End Link Worker study) as well as in developing and trialling research‐driven interventions led by academics (as in the case of CARE Plus). These two approaches are entirely in line with the new MRC guidelines on complex interventions, which for the first time have emphasized the importance of evaluation of ongoing interventions as well as RCTs.^9^

We hope the current paper has achieved its aims, but an obvious weakness was that our rapid reviews were not as rigorous as full systematic reviews would have been, and we may have missed some key publications. Nonetheless, our aim was to give an overview of how SDT has been used in recent studies in general, and specifically in complex interventions in the primary care setting. A further weakness was the limited patient and public involvement (PPI) input into the paper. Our funded research programme, of which this paper is an early output, has a newly embedded PPI group of four members with lived experience of long‐term conditions and/or of being informal carers. The group was not in place when this paper started but they did carefully read and commented on the draft manuscript and made useful suggestions on the text. They will be fully involved at all stages in the rest of the programme of research. In further developing the CARE Plus study using SDT as described, there will be full participation of a PPI group and specifically with patients living in deprived areas with lived experience of multimorbidity, building on our co‐design approach which has been a feature throughout the development of the intervention.^41^

## 5 CONCLUSIONS

The use of theory is an important consideration in primary care research and evaluation and is underutilized. SDT is a theory of motivation and basic psychological needs, little used yet in primary healthcare settings and multimorbidity. Its use to date suggests that it may be a useful candidate for theory‐informed research and evaluation in primary care and may be of particular importance in the development and evaluation of complex interventions for multimorbidity, given the growing clinical and economic importance of such patients globally, and the limited evidence‐base for the management of multimorbidity in primary care.

## CONFLICT OF INTERESTS

The authors declare that there are no conflicts of interest.

## AUTHOR CONTRIBUTIONS

All listed authors have contributed to the manuscript substantially and have agreed to the final submitted version. Huayi Huang and Stewart W. Mercer conceived of the paper and initially planned its content, which was discussed and agreed upon by all the other authors. Huayi Huang led the literature reviewing and wrote the first two drafts of the paper, which all the other authors commented on and revised in an iterative manner. Stewart W. Mercer substantially re‐wrote the paper for the penultimate version, which all the other authors commented on and Stewart W. Mercer revised for the final version which all other authors agreed to for submission.

## Supporting information

Supporting information.

## Data Availability

Data derived from public domain resources.
