# Supplementary material for: Using self‐determination theory in research and evaluation in primary care
Source: Health Expect. 2022 Oct 1;25(6):2700–8. doi: 10.1111/hex.13620 (PMC9700158; doi:10.1111/hex.13620)
Supplement: Supplementary file 1 — Supporting information. [file HEX-25--s001.docx]

**Supplementary file: Literature searches**

**For the search of recent reviews of SDT in health contexts,** we searched 10 bibliographic databases for relevant reviews and meta-analyses. 9 of the 10 were searched through Web of Science (see table S1 below); the other database searched was Medline. Date limits were from as far back as Jan 2016 (inclusive) to the 5th of August 2022. Documents retrieved were limited to *review* articles only – as defined by the indexing providing in Web of Science and Ovid. Figure S1 shows the flow of the records identified through the review process.

**Table S1: the searches for recent reviews of SDT in health contexts**

|  | **Search strategy** | **Number of Results Retrieved** |
| --- | --- | --- |
| Web of Science  *Web of Science Core Collection*  *BIOSIS Citation Index*  *Current Contents Connect*  *Data Citation Index*  *Derwent Innovations Index*  *KCI-Korean Journal Database*  *MEDLINE*  *SciELO Citation Index*  *Zoological Record* | 1. TI=("self-determination theory" or "self determination theory") AND TS=("health" or "healthcare")  *Limited to document type (“Review article”), and Publication Years 2016-2022 (inclusive)* | 12 |
| Ovid Interface  *Ovid MEDLINE(R) (1946 to July Week 5 2022)* | 1. ("self-determination theory" or "self determination theory").tw. | 1491 |
|  | 2. limit 1 to ("review articles" and yr="2016 -Current") | 43 |
|  | 3. ("health" or "healthcare").tw. | 2078347 |
|  | 4. limit 3 to ("review articles" and yr="2016 -Current") | 114709 |
|  | 5. 2 and 4 | 14 |

**Figure S1: Flow of records/reviews through the review process**

**Identification of reviews and meta-analyses via databases**

**Identification of studies via other methods**

Records removed *before screening*:

Duplicate records removed (n = 2)

Records identified from:

SDT website hosted by its proposers (n = 2)

Records identified from:

*Web of Science*

(all 9 databases, n = 12)

*Ovid Medline* (n = 14)

**Identification**

Records excluded:

Not specifically focused on SDT studies in health contexts (n = 10)

Not focused on reviews of studies of Patient or Caregiver Outcomes, e.g. instead reviewing Professional or 'App/Device Outcomes' (n = 3)

Is only protocol, not completed review (n=1)

Records screened

(n = 24)

Reports sought for retrieval

(n = 2)

Reports sought for retrieval

(n = 10)

Reports not retrieved:

No English fulltext (n = 1)

**Screening**

Eligible reports retrieved

(n = 2)

Eligible reports retrieved

(n = 9)

Reviews and Meta-analyses included (n = 11)

**Included**

**For the rapid review of studies using SDT in complex interventions in primary care settings,** 12 bibliographical databases were searched through the University of Edinburgh library services (Embase, Medline, PsycInfo, and all 9 from the Web of Science databases/collections; Table S2). No date limits were set. These searches are up to date as of 9^th^ August 2022. Figure S2 shows the flow of the 17 records identified through the review process supporting section 3.3 of the paper.

**Table S2: the searches for SDT use in complex interventions in primary care**

|  | **Search strategy** | **Number of Results Retrieved** |
| --- | --- | --- |
| Ovid Interface  *Embase (1980 to 2022 Week 31)*  *Ovid MEDLINE(R) (1946 to July Week 5 2022)*  *APA PsycInfo (1806 to August Week 1 2022)* | 1. ("self-determination theory" or "self determination theory").tw. | 8957 |
|  | 2. ("primary care" or "primary health care" or "primary healthcare" or "primary health" or "general practice$" or "general practise$" or "family medicine").tw. | 479879 |
|  | 3. "Compl$ Intervention$".tw. | 11041 |
|  | 4. 1 and 2 and 3 | 11 |
|  | 5. remove duplicates from 4 | 6 |
|  |  |  |
|  |  |  |
| Web of Science  *Web of Science Core Collection*  *BIOSIS Citation Index*  *Current Contents Connect*  *Data Citation Index*  *Derwent Innovations Index*  *KCI-Korean Journal Database*  *MEDLINE*  *SciELO Citation Index*  *Zoological Record* | 1. TI=("self-determination theory" or "self determination theory") | 1,493 |
|  | 2. TI=("primary care" or "primary health care" or "primary healthcare" or "primary health" or "general practice*" or "general practise*" or "family medicine") | 125,525 |
|  | 3. TI=("Compl* Intervention*") | 1,015 |
|  | 4. #1 AND #2 AND #3 | 0 |
|  | 5. #1 AND #3 | 1 |

**Figure S2: Flow of records/primary studies through the review process (section 3.3)**

**Identification of studies via other methods**

**Identification of studies via databases and registers**

Records identified from:

Citation searching (n = 1)

Team knowledge of relevant research activity in this area (n = 2)

Records removed *before screening*:

Duplicate records removed (n = 6)

Records identified from:

Embase (n = 6)

Medline (n = 4)

PsycInfo (n = 1)

Web of Science (n = 1)

**Identification**

Records screened

(n = 6)

Reports sought for retrieval

(n = 3)

Reports sought for retrieval

(n = 6)

**Screening**

Reports excluded:

Meeting/conference abstract (n = 2)

Eligible reports retrieved

(n = 3)

Reports assessed for eligibility

(n = 6)

Studies included in review

(n = 4)

Reports of included studies

(n = 7)

**Included**
